# Supporting stroke survivors to meet their personal rehabilitation needs in community-based arm rehabilitation: development of initial programme theories to explore what may work for whom, how and under what circumstances

**DOI:** 10.3389/fneur.2023.1089547

**Published:** 2023-06-02

**Authors:** Stefanie Schnabel, Frederike van Wijck, Lisa Kidd

**Affiliations:** ^1^School of Health and Life Sciences, Glasgow Caledonian University, Glasgow, United Kingdom; ^2^Alice Salomon University of Applied Sciences Berlin, Alice-Salomon-Platz, Berlin, Germany

**Keywords:** stroke, arm impairment, rehabilitation needs, self-management, realist informed analysis, community-based rehabilitation

## Abstract

**Objective:**

This study explored what worked for whom, how and under what circumstances in a community-based augmented arm rehabilitation programme that was designed to enable stroke survivors to meet their personal rehabilitation needs.

**Design:**

A mixed methods realist-informed study of data from a randomised controlled feasibility trial, comparing augmented arm rehabilitation after stroke with usual care. The analysis was designed to develop initial programme theories and refine these through triangulation of qualitative and quantitative trial data. Participants with a confirmed stroke diagnosis and stroke-related arm impairment were recruited from five health boards in Scotland. Only data from participants in the augmented group were analysed. The augmented intervention comprised evidence-based arm rehabilitation (27 additional hours over 6 weeks) including self-managed practice, and focused on individual rehabilitation needs identified through the Canadian Occupational Performance Measure (COPM). The COPM indicated to which extent rehabilitation needs were met following the intervention, the Action Research Arm Test provided data on changes in arm function, and qualitative interviews provided information about the context and potential mechanisms of action.

**Findings:**

Seventeen stroke survivors (11 males, age range 40–84 years, NIHSS median (IQR) 6 (8)) were included. Median (IQR) COPM Performance and Satisfaction scores (min.1-max.10) improved from pre-intervention 2 (5) to post-intervention 5 (7). Findings suggested that meeting rehabilitation needs was facilitated by strengthening participants’ sense of intrinsic motivation (through grounding exercises in everyday activities linked to valued life roles, and enabling them to overcome barriers to self-managed practice), and via therapeutic relationships (through trust and expertise, shared decision-making, encouragement and emotional support). Collectively, these mechanisms enabled stroke survivors to build confidence and gain mastery experience necessary to engage in new self-managed practice routines.

**Conclusion:**

This realist-informed study enabled the development of initial programme theories to explain how and in what circumstances the augmented arm rehabilitation intervention may have enabled participants to meet their personal rehabilitation needs. Encouraging participants’ sense of intrinsic motivation and building therapeutic relationships appeared instrumental. These initial programme theories require further testing, refinement, and integration with the wider literature.

## Introduction

Stroke is a major global health problem and responsible for a significant amount of long-term disability in adult life (1). It is estimated that three-quarters of stroke survivors initially experience arm impairment, and that only half of these regain full arm function within 6 months after stroke (2, 3). Moderate-quality evidence from a Cochrane overview of systematic reviews (4) showed a beneficial effect of at least 20 h of additional repetitive task practice on arm function, whilst strong evidence from a systematic review with meta-analysis (5) reported the benefits of at least 17 h of additional practice for arm motor function. However, according to these evidence syntheses, the minimum dose required tends to exceed the availability of routine health care, and therefore stroke survivors are required to undertake a substantial amount of self-managed practice (4, 6).

The *Early* VERsus *Later Augmented Physiotherapy compared with usual upper limb physiotherapy* (EVERLAP) study, a mixed methods, randomised controlled, multi-centre feasibility trial (Clinical trial registration number: ISRCTN 32522341), in which the current study is nested, focused on repetitive task-specific practice including therapist-led and self-managed practice. Given the limitations in the amount of face to face therapy that can be delivered in the UK’s National Health service, a core objective of the EVERLAP study focused on how self-managed practice of arm rehabilitation can be encouraged. Details about the EVERLAP study are reported elsewhere (7). Briefly, the study trialed the feasibility of comparing augmented arm rehabilitation (in addition to usual care) with usual care alone, provided within 9 weeks post stroke. Augmented arm rehabilitation consisted of 27 h of arm rehabilitation (45 min/day, 6 days/week over 6 weeks) in addition to usual care. It included therapist-led therapy (which was reduced during the six-week programme) and self-managed practice (which was increased over this period) (7) to improve meaningful functional activity involving the affected arm. The EVERLAP intervention comprised routine evidence-based treatment strategies (4, 5) for priming (e.g., mental practice), augmenting (e.g., functional electric stimulation) and practicing functional skills (e.g., repetitive task training). EVERLAP physiotherapists (referred to as ‘therapists’ henceforth) selected and progressed these treatment strategies according to individual participant-related factors (including pre-intervention arm severity, current best evidence (collated in a manual)) and their clinical judgement. Study participants could choose between three strategies to support their self-managed practice: a mobile phone reminder or a work booklet. The EVERLAP study focused on person-centred goals, operationalised using the Canadian Occupational Performance Measure (8) (COPM) to ensure that personal rehabilitation needs were addressed.

Enabling stroke survivors to meet their rehabilitation needs is a core aspect of rehabilitation, in which therapists have a role in helping stroke survivors to engage in goal setting, shared decision-making, and self-management (9, 10). However, an agreed definition of ‘rehabilitation need’ does not appear to exist (11). In the context of the current study, rehabilitation needs were defined as needs related to improving arm function, activity and participation as prioritised by individual stroke survivors on the COPM.

Most studies on intensive arm rehabilitation after stroke have concentrated on outcomes (4, 5, 12) with only a few studies investigating stroke survivors’ experiences of augmented arm rehabilitation (6, 13). Even fewer studies have explored for whom such interventions might work, how and under what circumstances. This insight is necessary - not only for clinical practice, but also in order to design better randomised controlled trials (RCTs) in stroke research (14).

Our previous qualitative paper reported how individual participants engaged with and embedded the learning from the EVERLAP rehabilitation into their daily routines (6). These findings revealed that whilst some individuals perceived that they had successfully met their rehabilitation needs, there were others who had not – but the reasons for these individual responses remained unclear. These findings suggest that before designing a definitive RCT, there is a need for a deeper exploration of the data to identify the hidden mechanisms through which the augmented intervention may have worked, for whom and how. This approach calls for a realist evaluation (15, 16) which, to our knowledge, has not yet been applied to arm rehabilitation programmes after stroke. Realist evaluation and realist synthesis are rooted in the realist paradigm (17, 18). Critical Realism (19) is centred between positivism and constructivism, distinguishing itself through its ontological and epistemological understanding of the world (20). In Critical Realism, programme theories are proposed to explain why and how an intervention programme may have caused the outcomes observed (16), using Context-Mechanism-Outcome configurations (CMOC) to structure the analysis (21). Context is observable and offers insights into the characteristics of the setting where the intervention takes place; mechanisms are the hidden concepts that, when uncovered, explain how intervention may or may not have worked; and outcomes are the observable intended or unintended effects of an intervention. By focusing on generative causation (i.e., the mechanisms by which the intervention works in a particular context leading to an outcome (22–24), a realist-informed study (25) enables a deeper understanding of what works for whom, how and under what circumstances in any given complex healthcare intervention (15, 16).

Therefore, the aim of the current realist-informed study, which to our knowledge is the first of its kind, aimed to develop initial programme theories to explain how, for whom and in what circumstances the EVERLAP intervention may have enabled stroke survivors to meet their rehabilitation needs. These initial programme theories, which will require further development and testing within the wider literature, are a first step towards a better understanding of how this intervention, as an exemplar of other similar complex rehabilitation programmes that focus on practice, may enable patients to achieve their rehabilitation needs.

## Methodology

The aim of a realist evaluation is to develop programme theories and test those with realist interviews. The current study focused on theory development only, aiming to create initial programme theories from a secondary analysis of primary qualitative and quantitative data from the EVERLAP study, ready for testing in a future realist evaluation study. Based on previous work (25), this realist-informed study was guided by the RAMESES II reporting standards for realist evaluations (17). Retroductive and abductive theorising, core analytical tools of Critical Realism, was employed to identify and conceptualise potential mechanisms of action or change in an intervention (25). To achieve realist explanations, Pawson and Tilley propose the use of CMOC (21, 25), a heuristic commonly used in realist approaches. This helps to articulate what works for whom, how and under what circumstances - specifically the relationship between a context that triggers mechanisms into producing certain outcomes- and also provides a structure to analyse mixed methods data (22). A description of how the context, mechanism and outcome are defined in realist approaches is found in Table 1.

**Table 1:** Description of context, mechanism and outcome.

Context	Context is seen as an observable thing in relation to space, people, places and things (26). According to Greenhalgh and Manzano, context in the sense of a relational and dynamic process is not a static observable ‘thing’ but it influences the mechanism through which the intervention works (26). Seeing context as a relational and dynamic process in the current study may help to understand why an intervention works in one context but not in another. For example stroke survivors engaging in task-specific training in hospital (context) may not work so well as when they engage in task-specific training in their own environment (context)
Mechanism	Mechanisms are the hidden concepts that explain how interventions may or may not work. Dalkin et al. (27), argue for a disaggregation of resource and response within mechanisms to understand the difference between the resource that is offered by the intervention and the way in which it guides the response of the participants (27). Distinguishing between resource and response within a mechanisms helps to differentiate mechanisms from contexts (27). For example, stroke survivors having a desire to resume life roles is a resource and feeling a sense of intrinsic motivation to be self-disciplined to engage in self-managed practice in order to resume those roles is a response to the resource
Outcome	The outcome is the intended or unintended effect of an intervention. To understand the causal link between an intervention and the outcome it is necessary to understand the underlying mechanism that connects them and the context in which the intervention and the outcome occurs (28)

### Ethics

All participants who took part in the EVERLAP study had to meet the inclusion criteria as shown in Box 1 and provide written informed. In cases where participants had the capacity to provide consent but were unable to write, verbal consent was obtained in the presence of a witness (an individual not involved in the study), who then signed the consent form on behalf of the participant. Participants in the augmented intervention groups were asked to provide consent for their participation in a post-intervention interview after the end of the augmented arm rehabilitation programme. Carers who did not consent but were present during the interview were made aware that their contributions would not be included in the analysis of the findings.

Ethical approval was granted from the National Research Ethics Service (REC Reference 14/WS/1136), NHS Research and & Development department and Glasgow Caledonian University’s School of Health and Life Sciences Ethics Committee. The study was funded by the Charitable Trust of the Chartered Society of Physiotherapy (N/12/10) and the sponsor was Glasgow Caledonian University.

BOX 1. **Inclusion criteria for the participants who took part in the EVERLAP study.**

- Age ≥ 18 years
- Diagnosis of stroke confirmed by CT/MRI
- Stroke-related arm impairment (score 0–56 on the Action Research Arm Test, min. 0, max. 57 points) (29)
- Capable of undertaking the allocated physiotherapy intervention and adhering to the study protocol, as per judgement of the treating clinician
- Capacity to provide informed consent to participate in the study
- Living within the community services catchment area of a particular study centre.

### Data collection

In the EVERLAP study, quantitative and self-reported outcome data included the Action Research Arm Test (29, 30), Motricity Index (31), Grip force (32), COPM (8), Numerical Pain Rating Scale (33), Motor Activity Log (34), Stroke Impact Scale (35) and the Hospital Anxiety and Depression Scale (36). Participants’ stroke severity and arm capacity were measured with the National Institute of Health Stroke Scale and the Action Research Arm Test (29, 30), respectively.

For this secondary analysis, pre-intervention data from the augmented intervention groups were compared with their post-intervention data, to explore what had changed and for whom over the course of the intervention. The COPM was used as a tool to operationalise stroke survivors’ rehabilitation needs and to capture person-centred goals, which included the performance areas of self-care, productivity and leisure (8). Within each performance area, stroke survivors selected their prioritised activity and rated their performance and satisfaction on each activity on a scale from 1 to 10 (1 being the lowest and 10 the highest score) (8). The minimal clinical important difference, which is +2 points (8), was applied to detect clinically meaningful improvements in the COPM between pre- and post-intervention and to identify those who responded to the treatment.

Quantitative outcome data were collected by the EVERLAP study physiotherapists. Interviews with stroke survivors who completed the EVERLAP augmented intervention were conducted by two researchers (including the first author), who were not involved in intervention delivery. The methodology for the interviews is reported in detail elsewhere (6). Briefly, semi-structured interviews with stroke survivors and their carers (if present) took place in stroke survivors’ homes, at the University or in hospitals following a topic guide – these were not realist interviews. The topic guide was developed based on *Normalisation Process Theory* (37, 38), and incorporated its four main constructs, i.e., coherence, cognitive participation, collective action and reflexive monitoring.

### Realist-informed data analysis

According to the RAMESES guidelines, initial programme theories can be developed from different sources (17) and can be used solely or in combination with another (39, 40). Qualitative data from the EVERLAP interview study (6) were used in triangulation with the quantitative data listed above. A conceptual framework of mid-range theories (39) guided the initial programme theory development. Relevant literature on rehabilitation needs was reviewed prior to the initial programme theory phase to form “if…then…statements,” which subsequently informed programme theory development. Similar to previous studies which have utilized this particular approach (39, 41, 42), qualitative interview data and quantitative data were the main source for the CMOC coding. The current realist-informed study followed a deductive and inductive logic (43). In a first step, interview transcripts were read and coded for causal insight (see Supplementary material S1), which means that the data map onto a CMOC, or partially identify elements of context, mechanism or outcome that can be configured into a potential CMOC. Secondly, all relevant qualitative and quantitative data were summarized to help with the triangulation and abductive theorising (44). The familiarization with the data set helped to identify the first links between context and outcome. In the next step, the qualitative data were catalogued in a table to differentiate between intervention strategy, context, mechanism-resource, mechanism-response and outcome (see Supplementary material S2). The initial ideas, which were rooted in the data, were drafted (see Supplementary material S3). For each initial programme theory, CMOCs were created and supported with quotations from the interviews and quantitative data. The interviews and demographic data were used to identify the context of the stroke survivors and potential mechanisms of action. This was then linked to the COPM scores to generate hypotheses about the relationship between individuals’ goals, life roles and their response to treatment. The first author conducted the literature review on rehabilitation needs, the coding, the triangulation of qualitative and quantitative data, the cataloguing and the development of initial programme theories. Throughout the whole process LK and FvW were involved and all steps of the data analysis process were discussed in multiple rounds between the research team until agreement was reached.

Retroduction was applied, which involves constantly moving between data and abstract theories to theorise the hidden mechanisms (16, 44). Retroductive theorising starts with the intervention’s outcome and works backwards to identify the hidden mechanisms that may have caused the outcome. In retroduction, some of the hidden mechanisms are clearly located in the data and others are “if…then… statements” supported by the data but not entirely confirmed by them (25, 44). By reading the transcripts, patterns of pre-stroke live were detected that were supported by the data, thereby enabling ‘if…then…statements’ to be drafted. Patterns of pre-stroke lives included activities that stroke survivors where engaged before their stroke and which played a role in rehabilitation after their stroke.

During the identification of CMOCs, the literature was consulted to identify middle-range theories that supported and helped to explain and expand on the programme theories (16). Middle-range theories bridge the gap between high level ‘grand theories’ and empirical research and give insight into how *types* of interventions work in different *types* of context (45), leading to *types* of outcomes at a more generalizable level (46). Following the approach by Dalkin et al. (16) to building programme theories, middle-range theories were used in this study to theorise phenomena found in the data to explain for whom, how and under which circumstances the EVERLAP intervention appeared to have worked. A conceptual framework of mid-range theories was established on the basis of explanatory power (39). A purposive search was conducted and seven potential middle-range theories were identified that helped to explain the initial programme theories. Four middle-range theories were selected, through review and discussion within the research team (39), on the basis of their relevance, ability to explain the phenomena observed and the authors’ prior knowledge in the field, i.e.: *Self-determination Theory* (47)*, Social Cognitive Theory* (48)*, Relational Agency* (49, 50) and *Normalisation Process Theory* (37).

Briefly, *Self-determination Theory* was used to explain intrinsic motivation to engage in self-managed practice (47). Deci & Ryan (47) argue that three psychological needs: *autonomy, competence* and *relatedness* need to be satisfied to develop intrinsic motivation. *Social Cognitive Theory* helped to explain intrinsic motivation and the therapeutic relationship (48). According to Bandura (48) *mastery experience*, *vicarious experience*, *verbal persuasion* and *psychological state* are necessary to achieve self-efficacy*. Relational Agency* explored all aspects of social relations in a relational sense and helped to explain the phenomena of therapeutic relationships (49, 50). Burkitt (49) argues that agency in the context of relational sociology is manifested in social relations and that agency develops through emotional relatedness. Edwards (50) proposes that a relational approach can enhance professionalism and expertise through ‘a capacity for interpreting and approaching problems, for reading the environment, for drawing on the resources there [and] being a resource for others’ (p. 179). *Normalisation Process Theory* consists of four constructs, i.e., *coherence, cognitive participation, collective action* and *reflexive monitoring* (37). *Normalisation Process Theory* was helpful for explaining phenomena related to embedding self-managed practice into participants’ daily routines. For example, there was evidence that stroke survivors developed an understanding of the need for self-managed practice (*coherence*) and reflected on how their engagement in this enabled them to meet their rehabilitation needs (*reflexive monitoring*) (37). A more detailed description of the middle-range theories can be found in the Supplementary material S4. In the sections below, middle-range theories are reported along with the initial programme theories to increase the explanatory power of the study findings (17).

In a final step, the initial programme theories were discussed with one of the two therapists who was available, and she confirmed support for the initial programme theories and gave insight into how she perceived the interactions with the stroke survivors in the context of these theories.

## Findings

### Demographics of participants in the EVERLAP study

Stroke survivors were recruited for the EVERLAP study from five different hospitals in the West of Scotland.

A total of 39 stroke survivors and 10 carers were eligible to take part in the interviews. Twenty-two participants were not available for the interview for the following reasons: 3 were lost-to-follow-up, 10 withdrew from the intervention and 9 declined the interview. In the secondary realist-informed analysis reported on here, the transcripts from 17 stroke survivors (six females, age range 40–84 years) and five carers were included.

Table 2 shows the demographics for the stroke survivors who were included in the realist-informed analysis. The anonymity of all stroke survivors and their carers was protected by using false names for publication and presentation. The variation in initial stroke severity, pre-intervention arm capacity and COPM scores indicate that the realist-informed analysis comprised a degree of heterogeneity within the study population.

**Table 2:** Characteristics of study participants.

Characteristics Stroke survivors (pseudo-nyms)	Sex	Age (y)	Marital status	Occupational status at the time of the interview	Carer	Consenting carer interviewed	Type of stroke	NIHSS	ARAT pre-inter-vention	ARAT post-inter-vention	COPM pre-intervention Median (Range) P S	COPM post-intervention Median (Range) P S
Maureen	F	72	Married	Retired	Yes	No	H	2	57	57	5 (1–10) 5.5 (1–10)	8 (1–10) 8 (1–10)
Brigit	F	65	Single	Retired	No	No	I	13	42	52	7 (5–7) 6 (5–7)	8.5 (8–10) 9 (8–10)
Ross	M	63	Married	Retired	Yes	No	I	5	39	46	1 (1–3) 2 (1–3)	2 (1–4) 2 (1–3)
Simon	M	65	Single	Retired	Yes	Yes	H	13	0	0	7 (7–7) 6 (6–6)	5 (5–5) 5 (5–5)
Anthony	M	56	Married	Employed	Yes	Yes	I	5	41	50	1 (1–5) 1 (1–3)	8 (7–8) 8 (5–8)
Timo	M	49	Married	Off sick	Yes	Yes	H	9	3	11	1 (1–1) 2 (2–2)	5.5 (3–8) 6.5 (5–8)
Lewis	M	75	Married	Retired	Yes	No	I	10	45	49	5.5 (2–8) 5.5 (2–8)	4 (1–8) 4 (1–8)
Jackie	F	82	Married	Retired	Yes	No	I	13	0	0	1 (1–1) 1 (1–5)	4 (1–5) 1 (1–4)
Chris	M	56	Single	Unemployed	No	No	I	1	57	57	4 (1–6) 6 (1–6)	5 (4–7) 6 (4–7)
Lydia	F	40	Married	Off sick	Yes	No	I	8	0	4	1 (1–2) 1 (1–1)	5 (2–7) 5 (1–5)
Sean	M	68	Has partner	Employed	Yes	Yes	I	4	52	53	5 (3–5) 5 (2–5)	9 (5–9) 9 (6–9)
Alex	M	73	Has partner	Retired	Yes	No	I	0	48	55	6.5 (6–7) 8.5 (1–10)	1 (1–3) 1 (1–1)
Peter	M	64	Married	Retired	Yes	No	I	3	57	57	9 (2–10) 9 (2–10)	7 (1–8) 7 (1–8)
Janet	F	74	Single	Retired	Yes	Yes	I	3	14	57	1.5 (1–3) 2 (1–4)	8.5 (1–10) 8 (1–10)
Thomas	M	72	Has partner	Retired	No	No	I	10	3	3	1 (1–10) 1 (1–8)	1 (1–1) 1 (1–1)
Danny	M	84	Widower	Retired	No	No	I	12	3	3	1 (1–1) 1 (1–1)	5.5 (1–6) 5 (1–6)
Lyn	F	76	Married	Retired	Yes	No	I	6	0	No data	1 (1–2) 1 (1–1)	No data

Overview of demographics, clinical characteristics and pre-intervention and post-intervention data of the stroke survivors included in interviews (names are false); F, Female; M, Male; H, Haemorraghic; I, Ischaemic; ARAT, Action Research Arm Test which measures upper limb capacity, scores range from 0–57 (57 indicating normal performance); NIHSS, National Institute for Health Stroke Scale which evaluates the neurological status after stroke, scores range from 0–42 (0 indicating no abnormal neurological status after stroke); COPM, Canadian Occupational Performance Measure; P, performance; S, satisfaction scores range from 1 to 10 (10 indicating normal performance and satisfaction).

### Synthesis of programme and middle-range theories

#### Initial programme theory 1: intrinsic motivation

In this initial programme theory, it was postulated that stroke survivors who engaged more consistently in augmented arm rehabilitation including self-managed practice, built exercises and activities into their daily routine and were more likely to meet their personal rehabilitation needs. It was believed that stroke survivors who build exercises and activities into their daily routine may subsequently see the benefit and meaningfulness of their rehabilitation, and therefore feel intrinsically motivated and determined to engage in self-managed practice.

A quote from one of the participants exemplifies the nature of the programme theory:

“I get up at 6 o’clock every morning and do some exercises yeah… I’m a lot stronger than I gave myself credit for. I am more determined than I ever gave myself credit for.” (Anthony)

Three underlying CMOCs are presented which are related to participants’ intrinsic motivation to self-managed practice.

##### CMOC 1: resuming pre-stroke life roles

When stroke survivors with valued family or work commitments engaged in a routine of self-managed practice (context), then they were more likely to meet their rehabilitation goal of resuming their life roles (outcome). Stroke survivors’ desire to return to or fulfil their life roles (mech-resource) triggered a sense of intrinsic motivation and discipline (mech-response) to engage consistently in self-managed practice.

In the interviews, valued life roles described by participants included being a parent, grandparent, being employed and having hobbies. The interview data showed that stroke survivors with work commitments - but also those of retirement age with commitments and valued life roles - were more motivated to engage in self-managed practice even if it required effort and persistence. One stroke survivor, Peter, talked about his life roles as a grandparent and his motivation to return to pre-stroke life:

“… I took early retirement to help my Son and Daughter looked after my Grandchildren. I will be taking up responsibility for looking after them two days a week. [I will] play with them in the house, take them out and run about with them and play in parks and things like that. Take my elder granddaughter to Nursery, pick her up from Nursery. The other one [name of grandson] he is only a year old. … So that was one of the wee objectives that I set myself because I suppose I am the sort of the home-maker as it is now, if you like. I tend to do all the shopping and the making the meals and do all the domestic stuff like the cleaning and tidying and things like that. So, I wanted to get back to that.” (Peter)

In another example, Anthony reported how work was an important part of his pre-stroke life and how this, and the financial implications of not being able to work, influenced his motivation to establish a routine of self-managed practice to address his personal goals:

“My hand and my brain are my tools of work so I need this working … I can’t accept I’m not being able to write or type and that’s how I have to work on these. …. The self-motivation for me is to get back to work. … So, I have got cars to pay, I have got a house to keep I have got a boy to put through school and things like that. I need to get back to work and that’s my motivation. I’m a lot stronger than I gave myself credit for. I am more determined than I ever gave myself credit for.” (Anthony)

Anthony also reflected on how his engagement in exercise pre-stroke, and the sense of self-discipline that this instilled in him, was helpful for establishing and sustaining his engagement in rehabilitation to meet his personal needs after stroke:

“I used to try I am not saying I did it every morning but I used to try because of the TAE KWON DO I tended to get up and do something [before the stroke]. Just even kind of stretching to make sure the muscles are working nice and easy. But I am probably doing it more at the moment [after the stroke].” (Anthony)

In another example, one of the participants, Lydia, reported engaging in self-managed practice in order to be able to fully engage in her role as a mother,

“This month I have been looking after him [young son] myself so it [the strength] is building up.” (Lydia)

The quantitative data showed a positive response to treatment in some participants, which was connected to their motivation to return to or resume their life roles. For example, the two participants (Anthony and Lydia) noted here who were of working age and had family responsibilities, were highly motivated to resume their life roles and engaged in self-managed activities involving the affected arm. In both cases their performance and satisfaction ratings improved, indicating response to the intervention (Table 2). The one exception was Lydia, who was not satisfied with her performance of changing the baby post-intervention.

Interestingly, other stroke survivors who expressed a lack of valued pre-stroke life roles that they wished to return to did not report consistent engagement, or motivation to engage in, self-managed practice.

The notion of stroke survivors finding the motivation to engage in self-managed practice in order to resume their life roles is an example of *Normalisation Process Theory’s* (37) core principle *coherence* because stroke survivors framed self-management as being an important way to help them resume valued life roles.

*Self-determination Theory* (47), in particular the concept of *Autonomy,* explained participants’ intrinsic motivation to engage in self-managed practice to resume life roles. When stroke survivors are more involved in decision-making about their rehabilitation plan and goal setting, they may feel more in control of their plan and therefore are more likely to act autonomously.

The quote below shows evidence of one of the participant’s, Anthony’s, goal to resume his martial arts hobby TAE KWON DO, which had an impact on his autonomy and relatedness to his social environment:

“As I say, I set myself em... when I went back to the TAE KWON DO at the start of each year my Instructor would ask us all what our goals are for the forthcoming year at the TAE KWON DO… And they said “what are yours” and I said “I have got three” [laughter]”. I says well “first one is to actually take part in a full class before the end of March but I think it is a tall order” The second one is “I am going to do a 5K before the end of July” … And the other one is to get, because I sat my grading my last grading in 2016 I am eligible to sit my next in 2019 so I want to be able to say at the end of the year, or my instructor to say at the end of the year, “you could maybe do it next year and we will consider you for it”.” (Anthony)

##### CMOC 2: grounding exercises in everyday activities

When therapists visited stroke survivors in their homes and invested time to find out about their everyday activities and their physical and social environments (context) then stroke survivors were more likely to engage in self-managed practice to meet their rehabilitation goals (outcome). This was believed to be because with knowledge and understanding of the context of stroke survivors’ daily lives, therapists could ground exercises and rehabilitation tasks in, and tailor their support to, the day to day activities that stroke survivors engaged in (mech-resource). Subsequently, this may have increased their sense of intrinsic motivation and engagement in self-managed practice (mech-response).

This approach is linked to *Self-determination Theory* (47) because it enabled stroke survivors to work autonomously on activities that held meaning within the context of their daily routines. Furthermore, practicing everyday tasks also created a feeling of *relatedness* to individuals’ social environment because they linked to their social environment, such as preparing meals for a partner or child. In turn, this may have motivated stroke survivors to engage more in their rehabilitation. This can be seen in the following example from Maureen:

“I enjoyed them coming and I felt it was much more practical than anything I received in the hospital… maybe because they [EVERLAP physiotherapists] were in my house and they knew what I was having to put up with.” (Maureen)

A carer reported on the EVERLAP exercises, which were tailored to the stroke survivors’ needs:

Carer: “…the actual activities increased, not severity, but it pushed him a bit further each time’. ‘[The exercises] were very focused on him.” (Carer of Timo)

Maureen reflected that the knowledge and understanding of her home environment that the therapists gleaned through home visits meant that the intervention could be tailored to routine activities, such as playing the piano and working in the kitchen, that were important to her sense of identity. Reflecting on her inpatient stay, she commented that she did not feel motivated by the usual care rehabilitation experience at that point because the activities were not tailored to the everyday activities that were important to her, which she describes in the following quote:

[EVERLAP was] much more helpful than the exercises I had at the hospital…. much more practical in a way. … it [in the hospital] was like functioning in a vacuum it didn’t relate to anything I was doing. (Maureen)

In this case, carry-over from intervention to daily activities was hampered.

The notion of stroke survivors finding the motivation to engage in self-managed practice because it relates to their daily lives aligns with the *Normalisation Process Theory* (37) constructs of *coherence* and *reflexive monitoring. Coherence* is demonstrated through stroke survivors’ understanding of their rehabilitation needs and what is required to manage their arm impairment. Reflecting on their engagement in activities and understanding how these are meaningful in the context of their daily lives, and how this reinforces their sense of identity demonstrates *reflexive monitoring*.

##### CMOC 3: pychosocial barriers to self-managed practice

This CMOC explores an inhibiting context and mechanism for engaging in self-managed practice.

Stroke survivors who reported depression or fatigue (context) may have found it more difficult to engage in self-managed practice to meet their rehabilitation goals (outcome). Depression or fatigue may have hindered them to develop a sense of mastery over building and maintaining their own practice routine (mech-resource). As a result, this may have failed to trigger a sense of intrinsic motivation (mech-response) to engage in self-managed practice.

Some of the participants in the study spoke of depression, mood swings, ‘laziness’ and fatigue. One participant, Alex, reflected on his experiences of this and how it affected his emotions and motivation to engage in rehabilitation:

“I had to get my Doctor to come out yesterday and he prescribed me Temazepam 50mg he thinks I am depressed, a bit depressed. I feel as if I am [depressed] because a lot of the times it’s happening with me they are getting on top of me. …it [tiredness] brings me down a bit and if it brings me down I don’t think I am going to be doing exercises. Because I think to do exercises you need to be feeling good within yourself to start doing them so that you can get the benefit from it.” (Alex)

Anxiety and depression can affect peoples’ levels of self-efficacy beliefs according to the *Social Cognitive Theory* (48). Therefore, stroke survivors with depression may need additional help such as *verbal persuasion* from health professionals or social networks to start engaging in a *mastery experience*.

**Figure 1:**
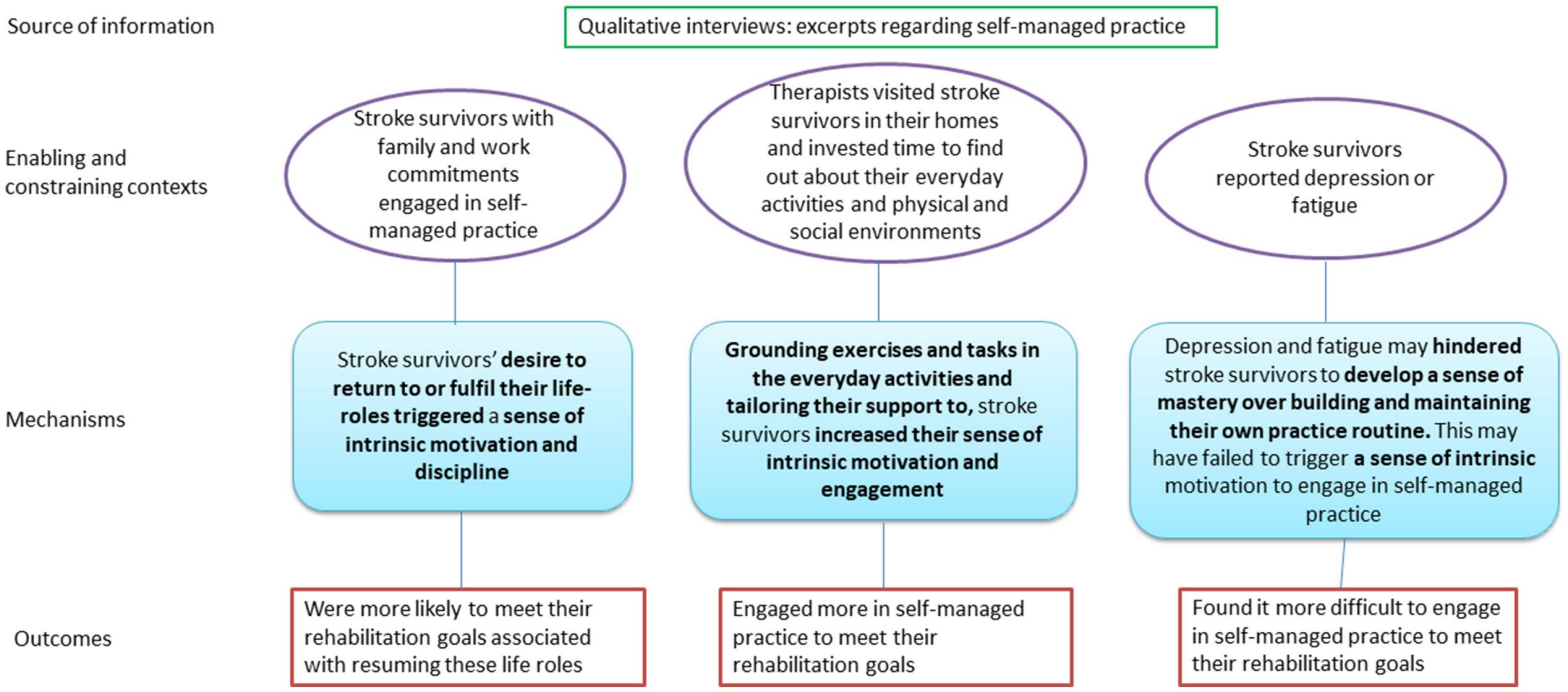
Initial programme theory 1: intrinsic motivation.

A summary of the underlying theories for programme theory *intrinsic motivation* is shown here (Figure 1).

#### Initial programme theory 2: therapeutic relationship

The second initial programme theory postulated that the supportive relationship and human contact between stroke survivors and therapists helped stroke survivors to meet their rehabilitation needs because they felt more confident and felt encouraged to engage in rehabilitation including self-managed practice.

By way of an example, a quote from one of the participants, Peter, reflects the essence for this programme theory:

“So that and em.... just the supportive nature of the EVERLAP physiotherapist coming out and the other EVERLAP physiotherapist when I saw her when I went to the University. … So, I suppose the human contact and the supportive relationship as I saw it was important and helpful to me.” (Peter)

Four underlying CMOCs comprise initial programme theory 2.

##### CMOC 1: trust and value of therapeutic expertise

When therapists demonstrated professionalism through their depth of knowledge and showed their commitment to working with stroke survivors (context), stroke survivors were more likely to engage in rehabilitation to meet their rehabilitation goals (outcome). Stroke survivors valued the therapists’ expertise (mech-resource) and as a result, this created a sense of trust (mech-response) which increased their engagement in self-managed practice.

The commitment from the therapists and their expertise were mentioned and valued by all participants. This helped stroke survivors feel supported, valued and listened to, and enabled them to build a trusting relationship with the therapists:

And I don’t think we can speak too highly of The EVERLAP physiotherapist. Carer: She was wonderful. Committed really, really knows what she’s talking about [laughter]. (Timo and Carer of Timo)

Another participant, Peter, stated that therapy input provided a space to talk about any problems:

“… it was just good to have somebody to talk to sometimes you could share, I know it is not the main element of the study, but to share the problems I was having with my left arm with this impingement it was good to talk to them because they were able to give me advice and things like that.” (Peter)

Valuing and trusting professional expertise can be aligned with a number of different theories. Firstly, *Relational Agency* (50), because therapists were able to interpret and approach individual stroke survivors’ problems and focus on the capacities and resources available to them. It also aligns to the concept of *relatedness* within *Self-determination Theory* (47) because it refers to the security and sense of safety that arose from the relationship with the health professionals, which increased stroke survivors’ intrinsic motivation to engage in self-managed practice. Finally, *collective action* within *Normalisation Process Theory* (37) reflects a sense of the relational integrity between stroke survivors and the therapists when working on rehabilitation goals, as well as building accountability and maintaining confidence and trust in working together.

##### CMOC 2: shared decision-making

When stroke survivors engaged in shared decision-making and therapists understood their priorities (context) then they were more likely to return to their everyday activities, which were formulated in the COPM (outcome). By making decisions about and having a sense of personal control over their rehabilitation plans (mech-resource) they may have been more committed and determined (mech-response) to engage in arm rehabilitation to meet their own goals.

All participants reported that they formulated their rehabilitation goals in cooperation with the therapists. One participant, Maureen, described her involvement in the rehabilitation plan and her commitment and determination to improve hand function:

“… with the EVERLAP physiotherapists I was very much involved in what they were doing. … Particularly with this left hand I need to make it stronger, I need to make it much more precise in what it does. And I would say that the same with the arm em... the pointing things like the fridge [exercise] they really help, they do. I am a control freak just so that you know I can’t help it….” (Maureen)

This is an example of *Relational Agency* (50) because of the interaction between stroke survivors and therapists during shared decision-making. The therapists were aware of each individual’s resources and personal environments, and supported the stroke survivor in their decision-making. Person-centred goal setting is at the heart of Deci and Ryan’s *Self-determination Theory* (47) and shared decision-making, as part of person-centred goal setting, is a positive determinant for motivation to engage in behavior change. *Autonomy*, *relatedness* and *competence* are essential for individuals to engage in person-centred goal setting. Once stroke survivors’ relationship with therapists had been established, they were likely to be more confident, motivated and *autonomous* in trying new exercises and activities by themselves. Maureen’s quote about being a ‘control freak’ suggests that she acted *autonomously* and *competently* and was therefore more involved in decision-making about her rehabilitation plan. She had the confidence to try out arm exercises, which created opportunities for further progression and self-discovery.

Person-centred goal setting aligns with *reflexive monitoring* and *collective action* as part of *Normalisation Process Theory* (37) because it encourages stroke survivors to reflect on their rehabilitation needs and, together with health professionals, decide on an appropriate and meaningful rehabilitation plan. Support from a competent and empathic health professional may help stroke survivors gain confidence to engage in decision-making about their rehabilitation plan focused on their individual needs.

##### CMOC 3: encouragement

When therapists purposefully took the time to get to know and understand stroke survivors (context) then they were more likely to engage in rehabilitation, including self-managed practice, to meet their rehabilitation goals (outcome). The encouragement from the therapists, tailored to the needs of individual stroke survivors, helped them to gain a better understanding about their capabilities (mech-resource). This enabled stroke survivors to gain a sense of mastery within their capabilities (mech-response) which then boosted their confidence and encouraged them to engage in problem solving and self-managed practice.

This statement is supported by the following quote:

“… if Timo was having a down time and because the stroke wipes him out at times, she would say “right let’s look at this” and it was problem solving “look at this set of activities that you were doing maybe standing up, let’s do them with you lying in a position where you are not fighting against gravity” and things like that.” (Carer of Timo)

Timo’s carer reported that the therapists were able to identify and resolve problems by working out what mental and physical resources were available to Timo. They encouraged him to try out alternative positions in which Timo was able to successfully complete the activities. This may have improved his confidence and enabled him to gain a sense of *mastery* as described by Bandura (48).

One participant, Alex, talked about becoming more confident through managing to achieve and engage in his rehabilitation exercises and how he felt about his *mastery experience*:

“I think them [the EVERLAP physiotherapists] coming gave me a lot more confidence. Because they have a good way with them. … [The exercises] give you more confidence I think and I was to do that… The good thing about them [the exercises] is managing to do it. It gave me great satisfaction, it gave me a boost. That was fine as per I know I can do them again and not feel apprehensive at all, you know. … Because I think with the illness you have got to feel confident, if you don’t feel confident you won’t do anything, you know. … The small ball bearing when I started to master that I felt really, really good. That gave me a good feeling.” (Alex)

Encouragement from therapists generated confidence to engage in independent practice, which aligns with the principles of *verbal persuasion* and *mastery experience* within Bandura’s *Social Cognitive Theory* (48). According to Bandura (48), *verbal persuasion* is required to increase a person’s trust in their abilities to master a task. The quotes shown throughout the previous and current sections provide evidence that *verbal persuasion* was used by the therapists; this provided stroke survivors with the positive support they needed to feel confident and gain an initial mastery experience. *Mastery experience* here refers to stroke survivors’ experience of competence in performing specific exercises or activities. Feeling competent to achieve or successfully engage in a task promotes a feeling of confidence, which can be helpful in encouraging stroke survivors to set new goals and to progress to more advanced exercises and activities, developing a sense of autonomy and mastery as they do so.

Encouragement from therapists may also be explained through *Relational Agency* because, according to Burkitt (49), social relations are based on emotional relatedness. Encouraging stroke survivors to engage in the intervention was facilitated by the therapists’ capacity to relate to individuals and ‘be a resource’ (50) for them, enabling them to build their confidence and develop a sense of *mastery experience*. The encouragement from the therapists also aligns with *collective action* as part of *Normalisation Process Theory* (37) as it emphasises the importance of collaboration with the therapists to achieve mastery experience. Additionally, *reflexive monitoring* describes how stroke survivors reflected on their progress and their developing competence and confidence, leading to a sense of achievement and mastery.

##### CMOC 4: emotional support

When therapists visited stroke survivors in their homes and provided a positive atmosphere through supportive and open communication (context) to support stroke survivors, then they were more likely to engage in rehabilitation including self-managed practice to meet their rehabilitation needs (outcome). The therapists provided emotional support through positive reinforcement and established a sense of rapport with stroke survivors (mech-resource). As a result, stroke survivors felt more confident and able to achieve their goals and overcome barriers during their rehabilitation (mech-response).

Through a sociological lens, emotional support offered as part of the therapeutic relationship may be seen as a relational construct, as communication and rapport between health professionals and stroke survivors happen in a social context (49).

In the following example, Lydia discussed the role of positive feedback and reinforcement by the therapists in building her confidence, particularly when feeling low mood:

“…the EVERLAP physiotherapist was a positive influence on me. When I was em … feeling down em … she said like I am doing really well and stuff…” (Lydia)

Another participant, Alex, reported an improvement in his confidence through establishing a sense of rapport with the therapists which allowed him the permission to be himself:

Interviewer: “OK did it maybe influence your mood when they were here in either a positive or negative way?”

Alex: “It was positive yeah”

Interviewer: “Can you tell me something about that?”

Alex: “Well I couldn’t be specific but I just know I felt comfortable within myself being able to have a laugh and a joke, it made me feel good…. I think them [EVERLAP physiotherapists] coming gave me a lot more confidence. Because they have a good way with them.”

The quotes show that emotional support can be provided through supportive communication and rapport, and that both resulted in increased confidence. As evident in the quotes, Lydia and Alex may have needed more emotional support through positive reinforcement and rapport compared to others in the study, because they had experienced fluctuations in their mood post stroke. Emotional relatedness as part of *Relational Agency* (49) created an opportunity for stroke survivors to build confidence and gain mastery experience. The therapists provided emotional support when problems arose and played a role in encouraging stroke survivors to engage in the intervention.

**Figure 2:**
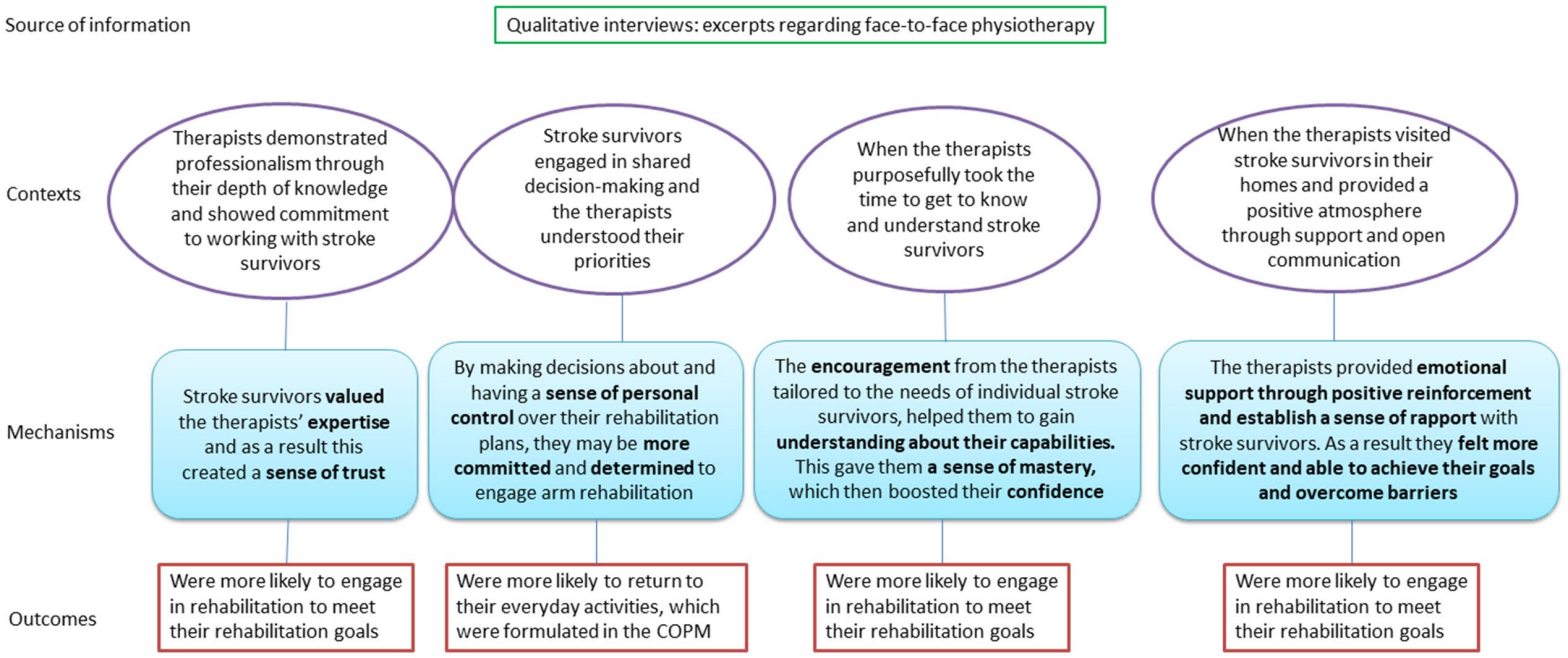
Initial programme theory 2: therapeutic relationship.

A summary of the underlying theories for programme theory *therapeutic relationship* is provided in Figure 2.

## Discussion

This realist-informed study generated initial hypotheses about how engaging with rehabilitation, including self-managed practice within the EVERLAP intervention study, may have addressed rehabilitation needs of people with impaired arm function after stroke, for whom and why. In summary, initial programme theory one, *intrinsic motivation,* suggests that focusing rehabilitation on valued life roles and relevant everyday activities within a meaningful social and physical context, and addressing psychosocial barriers enhanced an individual’s intrinsic motivation to engage in rehabilitation. These mechanisms seemed to work particularly well for stroke survivors with family and work commitments, those living in an environment they were already familiar with, and those who experienced fatigue and depression. Initial programme theory two, *therapeutic relationship,* suggests that the therapeutic relationship, which was underpinned by therapists’ expertise, a commitment to shared decision-making, encouragement and emotional support, was valued by stroke survivors. This helped them to feel a sense of trust in the therapists, confidence in themselves, and helped them to feel a sense of mastery over engaging in activities to address their rehabilitation needs. These mechanisms were enhanced when therapists were committed to working collaboratively with stroke survivors, understood their priorities, took time to get to know them and what mattered to them, and communicated openly and genuinely with stroke survivors. Those who already had intrinsic motivation to engage in self-managed practice also saw the support from and human contact with the therapists as invaluable. Both programme theories developed in this study applied to study participants presenting with a wide range in arm capacity and rehabilitation needs pre-intervention, including some for whom independent functional task practice would have been challenging.

### Resuming life roles as a trigger for intrinsic motivation

The findings of the current study suggest that the desire by some of the stroke survivors to resume valued life roles triggered a sense of intrinsic motivation to be self-disciplined to engage in self-managed practice in order to meet their rehabilitation needs. Self-discipline is aligned with traits such as self-control and self-regulation, and is associated with goal attainment (51, 52). The desire to resume life roles post stroke stems from the experience of a disruption to their identities, routines and age-appropriate life roles such as employment or parenting (53–56).. In the current study, stroke survivors also found that the stroke had caused a disruption to their daily activities and hobbies such as managing household duties, travelling and attending social events. In their study on needs and priorities of working age stroke survivors and their families Lawrence et al. (57) found that a commonly desired rehabilitation outcome was to return to pre-stroke roles. Morris et al. (58) also found that stroke survivors of working age were often motivated to engage in physical activity, which stemmed from the goal to return to their pre-stroke life and fulfilment of their life roles. Although physical activity is different to the arm-focused intervention in the current study, the common denominator is the element of repetitive practice as a vehicle to meet rehabilitation needs.

Findings from these studies resonate with those in the current study, which found that stroke survivors with family and work commitments and with a high degree of impairment saw specific exercises and activities as a way to help their recovery, and were motivated by their goal to return to pre-stroke roles, aligning with *autonomy* as part of *Self-determination Theory* (47) and *coherence* within *Normalisation Process Theory* (37).

### Activities of daily living trigger intrinsic motivation

Stroke survivors’ intrinsic motivation for and engagement in self-managed practice was linked to the fact that the exercises and activities were grounded in the context of their individual everyday activities. This has been supported in other stroke research (59, 60) Satink et al. (59) found that confidence in self-management develops through engaging in daily, routine activities, which gave stroke survivors a sense of mastery and perceived control. Regaining autonomy over their lives was empowering and developed stroke survivors’ sense of human agency (59, 60). The findings of the current study resonate with those of Satink et al. (59) and Kubina et al. (60), which showed that the psychological needs *autonomy* and *relatedness* within the theory of *Self-determination* (47) were addressed through embedding exercises into daily activities that were perceived as meaningful to stroke survivors.

Embedding exercises into daily activities can increase repetition, which – when completed successfully - boosts confidence to try out new activities so that these have potential to form a new habit for stroke survivors (61). Gardener and Rebar (62) suggest that once an activity becomes a habit, conscious motivation is no longer needed, and the habit is driven by a ‘context-cued impulse’. Grounding exercises in everyday activities that are meaningful to individuals (e.g., preparing meals for the family or driving a car to get to work), can therefore facilitate stroke survivors in resuming life roles that are based on habitual activities.

Repetitive practice of everyday activities may be a barrier however for those with psychological problems such as depression or fatigue. They are likely to see this as a burden, which, as the findings showed, may have an impact on motivation for and engagement in self-managed practice.

### Post-stroke depression as a barrier to intrinsic motivation

Post-stroke depression is a common problem and is often linked to reduced self-efficacy and poorer intervention outcomes (63). Stroke survivors with depression also tend to adopt more inefficient coping strategies whereas those with higher levels of self-efficacy tend to use more efficient coping strategies (63, 64). A study by Robinson-Smith, Johnston and Allen (65) found that stroke survivors who engaged in self-care that enhanced their sense of self-efficacy also experienced a positive impact on their depression. Those who had no occupation to go back to or lived alone reported more problems with depression (65), which resonates with the findings of the current study. What the current study highlights is the sense that depression can impact on self-efficacy and subsequent motivation and capacity to engage in rehabilitation including self-managed practice. Stroke survivors experiencing depression may therefore need additional support and commitment through *verbal persuasion* from the therapists to build self-efficacy and a sense of mastery in order to trigger intrinsic motivation to engage in rehabilitation.

### Therapists’ expertise

Stroke survivors in the current study expressed that they valued the therapists’ commitment and competence, which engendered a sense of trust, which in turn increased their engagement in self-managed practice. These findings are supported by a study by Doerfler and Kulnik (66) that found that a trusting client-therapist relationship entails showing commitment, shared decision-making and ‘to make an effort to get to know the person’ (p. 3631) (66). The systematic review by Killingback et al. (10) found that when using a self-management approach, physiotherapists liked to be seen as the expert with a desire to be in control. They also found that physiotherapists saw a “high-quality patient-therapist relationship” (p. 8) (10) characterized as being able to listen to patients and having good communication skills as central to working together with patients in a self-management approach (10). The current study did not find that the therapists had a desire to be in control - but rather perceived themselves to have a coaching role, ready to give advice and share expertise when it was requested by the stroke survivors. This aligns with *Relational Agency* (50) in terms of being a resource for others and *relatedness* as part of *Self-determination Theory* (47). The relational work stroke survivors do to build and maintain confidence and trust in their relationship with the therapists and the processes involved in engaging in shared decision-making link to *Collective action* within *Normalisation Process Theory* (37).

### Shared decision-making

The current study showed that stroke survivors who felt in control of their rehabilitation plan through shared decision-making were more committed and determined to engage in rehabilitation including self-managed practice. In the context of stroke rehabilitation, goal setting is used to facilitate shared decision-making, self-management and coping with life after stroke (9, 67). Shared decision-making involves stroke survivors and health professionals working together, with stroke survivors having the ultimate say in deciding their rehabilitation goals (68). Scobbie et al. (69) found that personal goal setting helped stroke survivors to resume their pre-stroke activities and reclaim their identity, which benefited their emotional wellbeing. The current findings resonate with those of Scobbie et al. (69) showing that personal goal setting in cooperation with the therapist helped stroke survivors to return to their pre-stroke activities including life roles and hobbies. Edwards’ (50) concept of *Relational Agency* offers an explanation because the therapists were aware of each individual’s resources and personal environments, and supported stroke survivors in their decision-making. The constructs of *autonomy, competence* and *relatedness* within *Self-Determination Theory* (47) can be aligned to the findings of the current study because stroke survivors need to form secure relationships with the physiotherapists and be involved in communication and shared decision-making to feel in control of their rehabilitation plan, so that rehabilitation activities and goals can be tailored to their needs.

### Encouragement and emotional support

The findings showed that encouragement from the therapists, which was tailored specifically to stroke survivors’ priorities, helped them to gain a better understanding of their capabilities. As a result, this enabled stroke survivors to gain a sense of mastery experience, which then enhanced their confidence and encouraged them to engage in self-managed practice independently. A study by Nott et al. (70) found that *mastery experience* and *verbal persuasion* were dominant concepts underlying self-management after stroke (70). The interviewees in that study discussed their increase in confidence through ‘doing’ activities and the encouragement they received from health professionals which had a positive impact on their outcomes (70). These findings support the current study findings, which showed the link between therapists providing encouragement and an increased sense of confidence amongst stroke survivors.

The current study also found that positive reinforcement from, and building a rapport with therapists provided emotional support to stroke survivors which helped them to gain confidence and a sense of mastery. This resonates with the findings from Horne et al. (71) in a study on the meaning of confidence after stroke. They found that confidence to engage in new activities often depended on the support from others involved, which reinforced the confidence in stroke survivors’ own abilities. Lawton et al. (72) also reported on a study with stroke survivors with aphasia, who felt a sense of connectedness with, and empathy from, therapists which fostered trust in their relationship. This is supported by the current study, which identified a relational approach as a foundation for the provision of emotional support in a therapeutic relationship.

The synthesised data show the importance of building and maintaining a therapeutic relationship for encouraging practice, because of the high value that was placed on the human contact and supportive relationships throughout the rehabilitation process. This also applied to those stroke survivors who were intrinsically motivated to engage in self-managed practice. Considering stroke survivors’ contexts including their life roles and needs to undertake specific everyday tasks triggered intrinsic motivation and self-discipline, and increased their engagement in the intervention to meet their rehabilitation needs. *Relational Agency* (49) offers an explanation for therapeutic relationships in that social relations are manifested in emotional relatedness, which can enable stroke survivors to develop their capacity for learning, confidence and a sense of mastery (7, 49). The current findings also align with *collective action* and *reflexive monitoring* as part of *Normalisation Process Theory* (37) because of the knowledge work and appraisal work that stroke survivors do to gain confidence to engage in further self-managed practice. Viewing the findings through the lens of *Social Cognitive Theory* (48), encouragement may be understood as *verbal persuasion* that gave stroke survivors the confidence to gain *mastery experience* and motivation to undertake further practice independently, which in turn enabled them to address their rehabilitation needs.

### Strengths and limitations

The study has several strengths and limitations. One limitation was that the initial programme theory development mainly used a data-driven approach. Although an initial literature review was undertaken, this was not a realist synthesis and therefore this approach may have missed what is already established in the literature that could potentially have created some valuable insights into the initial programme theories (38). A further limitation was that it was not known how self-reported engagement in self-managed practice aligned with actual engagement. Self-managed practice was not logged because a valid and feasible tool for this study population could not be identified (73). Logging self-managed practice would have given a more accurate record of the type and number of activities stroke survivors engaged in to meet their rehabilitation needs. Another limitation was that the therapists were specialised in and limited to providing augmented arm rehabilitation only and, being employed as part of the EVERLAP study, had more time with participants than in usual care. Therefore, the findings may not be directly transferable to stroke survivors receiving usual care. The selective sample is another limitation that may have implications for the findings since only 17 of the 39 participants in the original EVERLAP study agreed to take part in interviews about their experiences, so the data may not reflect the experiences of those who were not interviewed.

Despite the small sample, a realist-informed study (25) is a novel approach in stroke rehabilitation research. This allowed an in-depth analysis and the development of initial programme theories of what may have worked in the EVERLAP augmented intervention to support self-managed practice and address personal rehabilitation needs – and how. The EVERLAP study was a feasibility study for an RCT and therefore not designed to draw conclusions on intervention effects – nor was it designed as a realist evaluation study. The current study draws on Critical Realist theory and a realist logic model of enquiry to offer some explanation as to why the intervention may have been perceived by some individuals to have met some of their rehabilitation needs. The CMOCs, drawn from Pawson and Tilley’s (18) work and the heuristic more centrally used in realist syntheses and evaluation helped to unearth previously hidden mechanisms to better understand how and why stroke survivors engaged in self-managed practice. These initial programme theories and CMOCs now require further testing and integration with wider literature. Nonetheless, a strength of the current study is that it generated new hypotheses about how and why EVERLAP may have worked for some individuals, highlighting the importance of intrinsic motivation and the therapeutic relationship including building rapport, tailoring encouragement to individuals’ needs and developing stroke survivors’ confidence and sense of mastery in addressing their rehabilitation needs. Once these hypotheses have been tested, insights gained may help to inform future intervention design and randomised controlled trials of interventions in stroke rehabilitation that aim to address personal rehabilitation needs through rehabilitation that contains a component of self-managed practice.

### Implications for practice and research

The analysis reported on here aimed to develop initial programme theories to explain how the EVERLAP intervention may have enabled stroke survivors to meet their rehabilitation needs, and therefore any implications for practice are tentative. However, the findings emphasize the importance of a theory-driven approach to practice, drawing in particular on *Self-determination Theory* (47) and *Social Cognitive Theory* (48) to support stroke survivors to develop a sense of mastery and intrinsic motivation during their rehabilitation. This was shown to be necessary to enable them to develop new self-managed practice routines to enhance their recovery, which may be informed by *Normalisation Process Theory* (37, 38). The findings also highlight the importance of respectful and collaborative therapeutic relationships, underpinned by positive reinforcement, trust and rapport, to support people – especially those experiencing depression – to optimally engage in self-managed practice, which may be informed by *Relational Agency* (49, 50).

For future research, the initial programme theories developed here now require further testing and refinement in a realist evaluation study, and integration with the wider literature to enhance their validity, transferability, and level of theoretical abstraction. In particular, more research is needed to better understand how intrinsic motivation, self-discipline and building new routines can be enhanced through therapeutic input. This would seem to be relevant for long-term health conditions other than stroke (e.g., long Covid-19), where self-managed practice is central to optimizing recovery. Further research on building and maintaining therapeutic relationships in a self-management context is also needed, considering the ongoing global Covid-19 pandemic, where face-to-face contact with health professionals is being reduced. In this context, a deeper understanding is needed of which telehealth strategies work for whom, how and under which circumstances.

Through further empirical research, theory building and testing, programme theories could be developed for complex interventions that comprise self-managed practice (of which the EVERLAP intervention was an example) that could be applied more widely and inform clinical practice, education and service provision.

## Conclusion

This study used a novel realist-informed approach which was grounded in Critical Realism and used Context-Mechanism-Outcome configurations to explore for whom, how and under what circumstances an augmented arm rehabilitation programme, which included self-managed practice, had an impact on meeting personal rehabilitation needs. Despite the small sample and the need for further research, findings yielded new insights into how engagement in rehabilitation including self-managed practice can be enhanced through a focus on returning to valued life roles, developing a sense of confidence and mastery, and the provision of encouragement and emotional support.

These initial programme theories now require further testing and refinement in a realist evaluation study, and integration with the wider literature to enhance their validity and transferability to other stroke rehabilitation interventions involving self-managed practice.

## Data availability statement

The original contributions presented in the study are included in the article/Supplementary material, further inquiries can be directed to the corresponding author.

## Ethics statement

The studies involving human participants were reviewed and approved by Ethical approval was granted from the National Research Ethics Service (REC Reference 14/WS/1136), NHS Research and Development department and Glasgow Caledonian University’s School of Health and Life Sciences Ethics Committee. The patients/participants provided their written informed consent to participate in this study.

## Author contributions

SS collected qualitative data, undertook the data analysis and synthesis, and drafted the manuscript. FvW was the Principal Investigator of the EVERLAP study. LK provided the methodological expertise for this study. All authors contributed to analyzing and synthesizing the data and writing the article, and approved the submitted version.

## Funding

The authors express their thanks to the Chartered Society of Physiotherapy Charitable Trust for funding the EVERLAP study (grant number N/12/10) and Glasgow Caledonian University for funding a PhD studentship for SS.

## Conflict of interest

The authors declare that the research was conducted in the absence of any commercial or financial relationships that could be construed as a potential conflict of interest.

## Publisher’s note

All claims expressed in this article are solely those of the authors and do not necessarily represent those of their affiliated organizations, or those of the publisher, the editors and the reviewers. Any product that may be evaluated in this article, or claim that may be made by its manufacturer, is not guaranteed or endorsed by the publisher.
