# Supplementary material for: Supporting stroke survivors to meet their personal rehabilitation needs in community-based arm rehabilitation: development of initial programme theories to explore what may work for whom, how and under what circumstances
Source: Front Neurol. 2023 Jun 2;14:1089547. doi: 10.3389/fneur.2023.1089547 (PMC10272837; doi:10.3389/fneur.2023.1089547)
Supplement: Supplementary file 1 [file Data_Sheet_1.pdf]

Supplementary Material

Supplementary material 1

Coding strategy: code for causal insight

This is an example of excerpts of two interview transcripts, which were selected because of their causal insight, meaning that the data map into a Context-Mechanism-Outcome configuration or that it partially identifies with context, mechanism, outcome, which is then included in the Context-Mechanism-Outcome configuration.

Context	Mechanism	Outcome
'… just the **supportive nature of the EVERLAP physiotherapist** … it was just good to have somebody to talk to sometimes you could share, I know it is not the main element of the study, but to share the problems I was having with my left arm with this impingement it was good to talk to them because they were able to give me advice and things like that. (Peter) **[therapeutic relationship]**<br><br>**… I took early retirement to help my Son and Daughter looked after my Grandchildren. I will be taking up responsibility for looking after them two days a week.** [I will] play with them in the house, take them out and run about with them and play in	'…**through the EVERLAP Study I set myself some objectives** which are about things I was able to do before I had the Stroke which was doing housework, you know, dusting and doing dishes and ironing all the sort of domestic chores that everybody hates. So again I just made myself do them so that I would use both arms. '(Peter) [**Motivation, exercises related to everyday activities]**<br><br>'As I said a minute ago there, I wanted to do it (a) for my own benefit and participating in the Study but I didn't really need any Strategy **I am fairly focused and self-motivated guy.'** (Peter) **[intrinsic motivation]**	'I think they [the EVERLAP exercises] were good in that it **made me use the arm, my weaker arm, and it was good to just maintain the strength**.**' [exercises related to everyday activities]** (Peter)<br><br>'…you could always fit it (self-managed exercises) in because it only took maybe about half an hour to do them. So it's not a lot of time.' (Peter) **[engaging in self-managed practice]**<br><br>**So that was one of the wee objectives that I set myself because I suppose I am the sort of the home-maker as it is now, if you like. I tend to do all the shopping and the making the meals and do all the domestic stuff like the cleaning and tidying and things like that.**

parks and things like that. (Peter) **[resuming life roles]**<br><br>'She [the EVERLAP PT] was wonderful**. Committed really, really knows what she's talking about'** (carer of Timo) **[therapeutic expertise]**	'So the fact **that I was exercising I think was a motivating and positive factor for me** it gave me something to focus on and just I knew it was part of my recovery and would help me.' (Peter) **[intrinsic motivation]**<br>So I **suppose the human contact and the supportive relationship** as I saw it was important and helpful to me.' (Peter) **[therapeutic relationship]**<br><br>'The **EVERLAP physiotherapist was very very supportive** and didn't em... even if you don't feel like the exercising you know The EVERLAP physiotherapist is coming and em ...it is a good em... **she would just be very motivational**.' (Timo) **[therapeutic relationship]**<br><br>'It is the same as everything **you build up a relationship with the people you work with and that's very, very important**.' (Timo) **[trust, therapeutic relationship]**<br><br>**'We get out, we don't let the Stroke keep us in. A Stroke's there. Someone said to us, a good friend said "at the beginning initially the Stroke will manage you and you will learn to manage the Stroke" and that's true.'**	**So I wanted to get back to that**. (Peter) **[resuming life roles]**<br><br>'I don't like exercise but I have got to do it so I just try and **build on it to achieve my daily routine**.' (Timo) **[goal setting]**

	(carer of Timo) [**positive frame of mind, motivation]**<br><br>'… it was very much **a practical focus for the exercises** that Timo was doing. So that not only was he doing the exercises but **there was a purpose involved to perform the exercises**.' (carer of Timo) **[exercises related to everyday activities]**<br><br>'I think the actual exercises that The EVERLAP physiotherapist did to **support you** and showing you how to work if you were to.... em... what she did was really interesting because if Timo was having a down time and because the Stroke wipes him out at times, she would say "right let's look at this" and it was **problem solving**… it pushed Timo a bit further each time.' (carer of Timo) **[therapeutic expertise, encouragement]**	

Table 1: example of interview data of two participants coded to context, mechanism, outcome; first themes were identified in the square brackets

Supplementary material 2

Cataloguing all interview data into intervention strategy, context, mechanism and outcome in preparation for the hunches

Intervention Strategy	Context – enabling / constraining	Mechanism (Resource) (underlying programme theory)	Mechanism (Reasoning) (cognitive/emotional response)	Outcome
Problem solving<br><br>Setting objectives / goal setting<br><br>Augmented and specific arm exercises<br><br>Supported self-management<br><br>Face-to-face physiotherapy<br><br>Establish individual exercise routine<br><br>Mobile phone reminder<br><br>Physio session at the university<br><br>Home visits	Being occupied with EVERLAP exercises (self-managed) helped to keep active and prevent sitting too much<br><br>Supportive, motivational and positive atmosphere (EVERLAP PTs)<br><br>Structured and focused exercise programme<br><br>Engaging in exercises for the EVERLAP PT (they work hard, I should work equally hard)<br><br>Some couldn't work the mobile phone reminder<br><br>Exercising in a spare minute	Establish routine in exercising<br><br>Build relationship with PTs<br><br>EVERLAP PT being motivational and encouraging ('the EVERLAP PT coming is a motivator')<br><br>Importance of Human contact and supportive relationship with HPs<br><br>Positive frame of mind about stroke management<br><br>Practical focus of exercises	Self-determination<br><br>Confidence<br><br>Self-efficacy<br><br>EVERLAP helped to have a focus<br><br>Motivation comes from not accepting impairment<br><br>Motivation to see EVERLAP PTs at the university was to have someone to talk to<br><br>Self-motivated to return to pre-stroke life<br><br>Will<br><br>Being in control of rehabilitation success	Use weaker arm<br><br>Maintain the strength<br><br>Meeting COPM goals<br><br>Resume their life roles

EVERLAP exercises helped to use weaker arm<br><br>Practical exercises / task-specific exercises<br><br>Support network	Carers helping too much<br><br>Other health conditions prevented exercising or stroke management<br><br>Getting out of the house to do exercises and meeting people was a positive experience<br><br>Having someone to talk to about problems arising from the stroke was positive<br><br>Stopped exercising when EVERLAP programme finished<br><br>Rehab success depended on therapist<br><br>HPs encouraged stroke survivors to engage in exercising<br><br>Exercises related to life<br><br>Using compensation to deal with impairment	Exercising a motivating and positive factor (exercising keeps your mind off negative thoughts)<br><br>Knowledge on how to get better<br><br>Getting help from EVERLAP PTs lifted mood<br><br>Exercising helped to improve frame of mind<br><br>Time management<br><br>Having a positive mental attitude ('We get out, we don't let the Stroke keep us in. A Stroke's there. Someone said to us, a good friend said "at the beginning initially the Stroke will manage you and you will learn to manage the Stroke" and that's true.')<br><br>Commitment from the EVERLAP PT	Commitment<br><br>Trusted EVERLAP PTs<br><br>Awareness about using both arms (esp. if the affected side is the non-dominant side)<br><br>Not coping with all exercises<br><br>Carers prompting and encouraging to do exercises<br><br>Exercising has a positive impact on mood (having something to do)<br><br>Stopped exercises because of laziness or not doing any benefit<br><br>EVERLAP PTs being a positive influence<br><br>Encouragement form EVERLAP PTs helped to be motivated<br><br>Acceptance of the programme<br><br>Feeling comfortable within oneself and confident to start exercising	

	Acceptance of impairment<br><br>Depression<br><br>Going back to pre-stroke activities<br><br>Pre-stroke identity (work, hobbies, family)	Capability to work the mobile phone reminder<br><br>Motivational social network<br><br>More time with the PTs<br><br>Financial resources to get additional help<br><br>Wanting to get the hand better<br><br>Shared decision-making (PT and patient)<br><br>Need to be able to feel good within yourself to start doing exercises<br><br>Being confident to self-manage stroke<br><br>Going back to pre-stroke life (feeling useful again)<br><br>Guidance for carers on how to support stroke survivors	Confidence building through EVERLAP PT visits	

Table 2: Cataloguing the qualitative interview data into intervention strategy, context, mechanism and outcome

Supplementary material 3

Development of hunches: Data are categorised into themes, intervention strategy, context, outcome, mechanism resource and mechanism-response and drafted as if…then…statements

Themes	Intervention Strategy	Context	Outcome	Resource - Mechanism	Response - Mechanism	Full Statement
**Supported self-management**	Mobile phone reminder	Engagement in supported self-management	More likely to meet rehabilitation goals	To give the **choice** of engaging in exercises	In control over their engagement in supported self-management and rehabilitation progression	If stroke survivors engage in supported self-management then they are more likely to meet rehabilitation goals because the mobile phone reminder gives stroke survivors the choice of engaging in exercises or not and as a result they are more in control over their engagement in supported self-management and their rehabilitation progression.
**Supported self-management**	Booklet	Encouraged by the EVERLAP therapist to engage in self-management	More likely to meet their COPM goals	Choice of everyday exercises	Stroke survivors were autonomous in their choice of exercises they wished to practice according to their needs	If stroke survivors were encouraged by the EVERLAP therapists to engage in self-management then they were more likely to meet their COPM goals because the booklet provided a choice of everyday exercises and as a result, stroke survivors were autonomous in their choice of exercises they wished to practice according to their needs.
**Exercise routine**	Mobile phone reminder	Routine of exercising before the stroke	Establish an exercise routine and work on their	Mobile phone reminder helped to re-established an exercise routine	Self-motivated to return to their routine and meet rehabilitation goals	If stroke survivors had a routine of exercising before the stroke then they will find it easier to establish an exercise routine and work on their

			rehabilitation goals			rehabilitation goals because the mobile phone reminder as part of EVERLAP helped to re-establish an exercise routine and as a result, stroke survivors feel self-motivated to return to their routine and meet rehabilitation goals.
**Daily routine**	Task-specific practice	Encouraged to implement their exercises into their daily routine and use weaker arm	More likely to meet personal rehabilitation goals	Task specific practice gives them the opportunity to work with the weaker arm and practice everyday tasks	Gives them the confidence and independence to master their daily routine	If stroke survivors are encouraged to implement their exercises into their daily routine and use their weaker arm then they are more likely to meet their personal rehabilitation goals because task specific practice gives stroke survivors the opportunity to work with the weaker arm and practice everyday tasks and as a result this approach gives them the confidence and independence to master their daily routine.
**Life after stroke**	Task-specific exercises	Return to work	Improving skills (writing and typing) that are relevant for their work	EVERLAP exercises tailored to stroke survivors needs	More engagement because of the relevance of the task-specific exercises	If stroke survivors need to return to work after their stroke then they will focus on to improve the skills (writing and typing) that are relevant for their work because the EVERLAP exercises were tailored to stroke survivors needs and therefore focussing on the exercises that help with the skills necessary for their work leads to more engagement because of the relevance of the task-specific exercises.

**Support network**	Involvement of support network in rehabilitation	Support network, including relatives and the wider community	More likely to meet rehabilitation goals	Emotional and practical support from network	Stroke survivors gain confidence and encouragement to work on their goals and set more challenging goals	If there is a support network, which includes relatives and the wider community, available to stroke survivors then they are more likely to meet their rehabilitation goals because of the emotional and practical support they receive from their network and as a result, stroke survivors gain confidence and encouragement to work on their goals and may find it easier to set more challenging new goals.
**Goal setting**	Goal setting	Goal setting approach embedded in stroke survivors' ADLs and leisure activities	Rehabilitation needs will be met a lot quicker	Goals were tailored to their individual needs	Stroke survivors feel encouraged and disciplined to meet their personal rehabilitation goals	If a goal setting approach is embedded in stroke survivors' ADLs and leisure activities then rehabilitation needs will be met a lot quicker because goals within EVERLAP were tailored to their individual needs and therefore stroke survivors feel encouraged and disciplined to meet their personal rehabilitation goals.
**Depression**	Supported self-management	Depression diagnosed after stroke	More difficult to engage in supported self-management and therefore to achieve rehabilitation goals	Because of depression, less confident to self-manage	Require more help and support from therapists	If depression is diagnosed after stroke then it may be more difficult for stroke survivors to engage in supported self-management and therefore to achieve rehabilitation goals because if they feel depressed they will be less confident to self-manage and so they require more help and support from the therapists.
**Depression**	Therapeutic relationship	Stroke survivors who feel	May be able to meet their personal	Therapists may be more likely to encourage stroke	Stroke survivors are more confident and feel positive about	If stroke survivors who feel depressed and lack in confidence engage more in face-to-face therapy sessions then

		depressed and lack in confidence engage more in face-to-face sessions	rehabilitation goals	survivors to engage in rehabilitation	themselves when engaging in rehabilitation	they may be able to meet their personal rehabilitation goals because therapists may be more likely to encourage stroke survivors to engage in rehabilitation and as a result, stroke survivors are more confident and feel positive about themselves when engaging in rehabilitation.
**Motivation**	Therapeutic relationship	Stroke survivors lack intrinsic motivation to engage in rehabilitation	They need the help of therapists to improve in the activities which were chosen in the COPM	Therapists provide support and guidance	More likely to be motivated to engage in rehabilitation	If stroke survivors lack intrinsic motivation to engage in rehabilitation then they need the help of therapists to improve in the activities which were chosen in the COPM because therapists provide support and guidance and as a result, stroke survivors are more likely to be motivated to engage in rehabilitation.
**Motivation**	Supported Self-management	Believe in 'the more the better'	More likely to engage in self-management to meet their personal rehabilitation needs	A positive mind set about rehabilitation help stroke survivors to value it	Feeling of confidence and in control over rehabilitation progress and success	If stroke survivors believe in 'the more the better' then they are more likely to engage in self-management and meet their personal rehabilitation goals because a positive mind set about rehabilitation help stroke survivors to value it and therefore stroke survivors felt confident and in control over their rehabilitation progress and success.
**Treatment burden**	Supported self-management	Stroke survivors with other chronic health issues	More likely that they do not improve in their chosen goals	They may feel overwhelmed by the management of several health issues	Not able to prioritise and focus on their goals	If stroke survivors with other chronic health issues are expected to engage in supported self-management then it is more likely that they do not improve in their chosen goals as part

						of their rehabilitation plan because they may feel overwhelmed by the management of several health issues and therefore they are not able to priorities and focus on goals in their rehabilitation plan.

Table 3: Development of hunches

Supplementary material 4

Description of mid-range theories

Self-determination Theory, Deci & Ryan[47]	*Self-determination Theory* is used widely to explain motivation in health-related behavioural change. *Self-determination Theory* distinguishes between intrinsic and extrinsic motivation and argues that three psychological needs: *autonomy*, *competence* and *relatedness* need to be satisfied for a person to develop intrinsic motivation. Acting *autonomously* means there is willingness and volition involved in doing an activity. *Competence* relates to communication or feedback from healthcare professionals who have the relevant expertise, which increases the person's feeling of confidence and intrinsic motivation. *Relatedness* refers to the social environment, where relationships to others such as therapists or peer groups increase the person's intrinsic motivation.
Social Cognitive Theory, Bandura[48]	Confidence to engage in self-managed activities after stroke is closely linked to Bandura's *Social Cognitive Theory*. M*astery experience* describes a person's experience of competence to successfully engage in activities and to achieve goals. *Vicarious experience* describes the process of persons learning through observing others who engage in a task. *Verbal persuasion* refers to health professionals encouraging persons to engage more in activities and set goals. Barriers to engaging in activities including the *psychological state* (e.g. depression), which may hinder self-efficacy.
Relational Agency, Burkitt[49] and Edwards[50]	*Relational Agency* explores all aspects of social relations in a connected sense and helps to explain the therapeutic relationship. Burkitt[49] argues that agency in the context of relational sociology is manifested in social relations and that agency develops through our emotional relatedness. At the centre of his theory stands the concept of interdependence between humans, where relational interaction happens in different ways such as physical, emotional, practical, social, political, meaningful and economical. Edwards[50] in her work on relational agency proposes that a relational approach can enhance professionalism and expertise through 'a capacity for interpreting and approaching problems' (p. 179), focusing

	on the resources there and 'being a resource for others' (p. 179).
Normalistion Process Theory, May et al.[37]	*Normalisation Process Theory* is a framework to design and evaluate complex interventions. Set in the context of stroke and the components of the EVERLAP study, the four constructs of *Normalisation Process Theory* may be applied as follows: *coherence* is the sense-making work, which includes stroke survivors developing an understanding of their rehabilitation needs and the work they need to do to integrate the exercises or activities into their daily routine, and how they will do this as part of their self-managed practice. *Cognitive participation* is the relational work that stroke survivors, together with the healthcare professionals, do to build and maintain a routine around undertaking exercises or activities, as part of their self-managed practice. *Collective action* is operational work, which means that stroke survivors have confidence in the EVERLAP physiotherapists and their carers, and together engage in exercises or activities to meet their rehabilitation needs, as formulated in their COPM. *Relational integration* is one component of *collective action* and explores the knowledge work that stroke survivors do to build and maintain their confidence in engaging in self-managed arm rehabilitation, and in the therapeutic relationship during the rehabilitation. *Reflexive monitoring* is the appraisal work that stroke survivors do to understand and assess their engagement in exercises and activities, how well these help towards their recovery and meeting rehabilitation needs as formulated in the COPM.

Table 4: Description of middle-range theories: *Self-determination Theory*[47], *Social Cognitive Theory*[48], *Relational Agency*[49,50] and *Normalisation Process Theory*[37]
